# SESN2 correlates with advantageous prognosis in hepatocellular carcinoma

**DOI:** 10.1186/s13000-016-0591-2

**Published:** 2017-01-24

**Authors:** Shaosen Chen, Weigang Yan, Weiya Lang, Jing Yu, Li Xu, Xinyu Xu, Yunlong Liu, Hongguang Bao

**Affiliations:** 1 Department of Thoracic Surgery and Oncology, the No. 2 Affiliated Hospital of Qiqhar Medical University, Qiqhar, 161000 China; 2 Department of Oncology, the First Hospital of Qiqhar City, Qiqhar, 161000 China; 3 Department of Biochemistry and Molecular Biology, School of Basic Medical Sciences of Qiqhar Medical University, Qiqhar, 161000 China; 4 Department of Histology and Embryology, School of Basic Medical Sciences of Qiqhar Medical University, Qiqhar, 161000 China; 5 Department of Pathology, Jiangsu Cancer Hospital, Affiliated Cancer Hospital of Nanjing Medical University, Nanjing, 210000 China

**Keywords:** SESN2, qPCR, Western blotting, IHC, Hepatocellular carcinoma

## Abstract

**Background:**

SESN2 plays important roles in the regulation of cell survival, cell protection, and tumor suppression. However, the relationship between SESN2 expression and the clinicopathological attributes of hepatocellular carcinoma (HCC) is barely investigated.

**Methods:**

One-step quantitative reverse transcription PCR, Western blotting analysis in 15 fresh HCC tissues, and immunohistochemistry (IHC) analysis in a tissue microarray (TMA) containing 100 HCC cases were performed to examine SESN2 expression. Survival analyses by Cox regression method and Kaplan-Meier curve were performed to describe the overall survival of 100 HCC patients.

**Results:**

The SESN2 expression in HCC tissues declined dramatically compared with the corresponding noncancerous tissues, and SESN2 expression was remarkably associated with HBV infection (*p* = 0.019), HCV infection (*p* = 0.001), and lymph node metastasis (*p* = 0.033). Survival analysis further demonstrated that SESN2 expression could serve as an independent prognostic biomarker for overall survival in univariate (*p* = 0.001) and multivariate analyses (*p* = 0.003).

**Conclusion:**

The data are the first to indicate that SESN2 might be a novel prognostic marker for HCC and that elevated SESN2 expression predicts advantageous outcomes in HCC patients.

## Background

Hepatocellular carcinoma (HCC) represents the fifth most common cancer type and causes more than 500,000 cancer-related deaths every year worldwide [1–3]. Although the majority of HCC cases develop in Asia, cases in China account for more than half of initially diagnosed HCC patients all over the world, and the city of Qidong in East China is one of the most highly endemic areas for HCC [4, 5]. The etiology of HCC is diverse and complicated; hepatitis B (HBV) and hepatitis C (HCV) viral infections as well as liver cirrhosis often contribute to HCC development [6, 7]. Despite substantial improvements in HCC management, including surgical resection, microwave ablation, liver transplantation, radiofrequency and chemotherapy, over the last 10 years, the prognosis of HCC remains unsatisfactory; moreover, the 5-year overall survival rate is less than 30% [8, 9]. For now, alpha-fetoprotein (AFP) is still the most widely acknowledged marker in early detection and follow-up surveillance for HCC [10]. However, because of the number of AFP negative HCC patients and the inadequate understanding of the molecular mechanism of HCC tumorigenesis, studies focusing on the novel biomarkers that are involved in the carcinogenic process of HCC development and correlated with malignant characteristics of HCC are urgently needed [11].

SESN2, is transcriptionally regulated by p53, and belongs to the evolutionarily conserved sestrin family [12]. As a critical downstream effector of p53, SESN2 could be induced in a p53-dependent manner, such as with DNA damaging treatments, or in a p53-independent manner, including hypoxia or oxidative stress [12, 13]. SESN2 is involved in the regulation of cell survival, protection, and regeneration [14, 15]. Moreover, SESN2 acts as an important contributor in autophagy induction and tumor suppression [16, 17]. For example, SESN2 expression could be increased via the c-Jun N-terminal kinase (JNK) pathway to influence autophagy induction in cancer cells [9, 18]. Several gene expression analyses have also shown down-regulated SESN2 expression in different types of lung cancers [19, 20]. In addition, SESN2 expression inhibits cancer growth while increasing the sensitivity of cancer cells to ionizing radiation [15, 21]. The upregulation of SESN2 can also induce apoptosis through the p53 signaling pathway [22]. These studies suggest that SESN2 acts as a multi-functional molecule and is critically associated with tumor development. Nevertheless, the characteristics of SESN2 expression in HCC have been barely investigated.

To explore SESN2 expression in this study, we first detected the SESN2 expression using one-step quantitative-polymerase chain reaction (qPCR) test and Western blotting analysis in 15 fresh HCC tissues. Subsequently, we performed immunohistochemistry (IHC) analysis in a tissue microarray (TMA) containing 100 HCC tissue samples. Then, we analyzed the correlations of SESN2 expression with important clinicopathological characteristics of HCC patients. Finally we evaluated the prognostic role of SESN2 expression for HCC.

## Methods

### Tissue samples

Fifteen fresh HCC tissue samples and corresponding noncancerous tissue samples were collected from the Department of Surgery, the No.2 Affiliated Hospital of Qiqhar Medical University from May 2013 to Dec 2013. Simultaneously, 100 formalin-fixed, paraffin-embedded HCC and corresponding noncancerous tissue samples (each pair of HCC and corresponding noncancerous sample was from the same patient) were collected from the Department of Surgery, the No.2 Affiliated Hospital of Qiqhar Medical University (10 samples) and Xinchao Biotech Co., Ltd (90 samples) (Shanghai, China) for retrospective analyze. The HCC TMA from Xinchao Biotech Co., Ltd was described in a previous study [23]. No patients received chemotherapy, radio therapy or other immunotherapy before hepatic surgery. Substantial clinical data were also recorded in 2013, including gender, age, HBV infection, HCV infection, liver cirrhosis, tumor size etc. from medical records (10 samples) and purchased tissue microarray samples (90 samples). All 100 HCC samples of TMA were entered into the survival analyses. Clinical staging was classified according to the 2002 American Joint Committee on Cancer/International Union Against Cancer TNM staging system [24]. All enrolled patients signed written informed consent and all experimental procedures were implemented following the approved protocols of Qiqhar Medical University. All authors had access to identifying information during data collection.

### Detection of mRNA expression of SESN2 by one-step qPCR test

One-step qPCR test was performed to detect the mRNA expression of SESN2 preliminarily in 15 fresh HCC and corresponding noncancerous tissue samples following the protocols in our previous publication [25]. The primers for SESN2 were designed as follows: forward primer 5’ - AGA GGG CAC AGG AAA GAA-3’; reverse primer 5’-TCA AGC ATA AAG GAC CAA A-3’. The glyceraldehyde 3-phosphate dehydrogenase (GAPDH) was employed as internal control and the primers for GAPDH were as follows: forward primer 5’-TGC ACC ACC AAC TGC TTA GC-3’; reverse primer 5’-CTC ATG ACC ACA GTC CAT GCC-3’. SensiMixTM One-Step Kits (Quantace, Berlin, Germany) were purchased to execute qPCR test (Bio-Rad PCR system). The data of qPCR test were analyzed using the 2^-∆∆Ct^ method that was described in the previous studies [25, 26].

### Detection of protein expression of SESN2 by western blotting analysis

Total protein was isolated from 15 HCC and corresponding noncancerous tissue samples. The western blotting analysis was described in previous research [27]. Breifly, total protein was loaded, separated and transferred onto nitrocellulose membrane. The membranes were first incubated with primary mouse monoclonal anti-SESN2 antibody (1: 500, Abcam, ab57810, Cambridge, MA, USA) and secondary antibody (Boster, Wuhan, China). β-actin (Sigma, USA) was used as an internal control.

### Measurement of protein expression of SESN2 by IHC analysis

IHC analysis was executed to measure the protein expression of SESN2 in a TMA containing 100 HCC and corresponding noncancerous tissue samples following the protocols in a previous publication [27]. HCC TMAs were incubated with a primary mouse monoclonal anti-SESN2 antibody (1:250, Abcam, ab57810) and then a second anti-mouse horseradish peroxidase-conjugated antibody (Santa Cruz Biotechnology, Santa Cruz, CA, USA) was added. Phosphate-buffered saline (PBS) was used as negative control. The IHC result was calculated in light of the intensity and percentage of positive staining of SESN2 in HCC cells. The intensity of staining was categorized into 4 levels: level 0 = negative staining of SESN2, level 1 = weak positive staining of SESN2, level 2 = moderate positive staining of SESN2, level 3 = strong positive staining of SESN2. Similarly, the percentage of staining was also ranked into 4 grades: grade 1 = 0–10% percentage of positive SESN2 staining, grade 2 = 11–50% percentage of positive SESN2 staining, grade 3 = 51–80% percentage of positive SESN2 staining and grade 4 = 81–100% percentage of positive SESN2 staining. The product of the intensity level and percentage grade led to the decisive staining score. The final IHC result of SESN2 staining was defined by a two-level ranking type: <3 staining score suggests low SENS2 protein expression while 3–12 staining score suggests high SENS2 protein expression.

### Statistical analysis

The data of qPCR test and western blotting analysis was analyzed with the Student’s *t* test. The relationship between SESN2 expression and clinicopathological factors of HCC patients was analyzed by chi-square test. Survival analysis was accomplished using Cox’s regression models. The Kaplan-Meier curve was drawn to determine independent prognostic factors for HCC patients. The level of significance was set at *p* < 0.05. All data were analyzed with SPSS 16.0 (SPSS Inc, Chicago, IL, USA).

## Results

### Detection of mRNA expression of SESN2 by one-step qPCR test

**Fig. 1:**
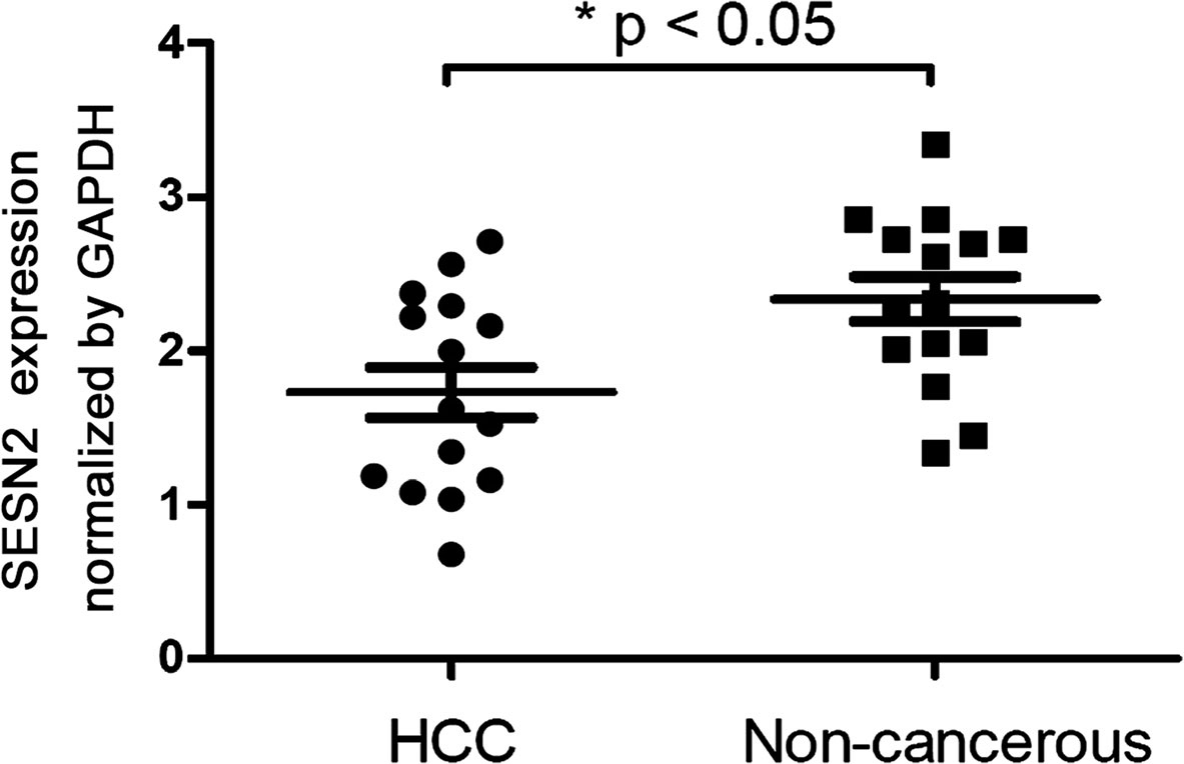
One-step quantitative real-time polymerase chain reaction (qPCR) test was performed to examine SESN2 expression in hepatocellular carcinoma (HCC) and non-cancerous tissues. The SESN2 mRNA expression was statistically lower in HCC tissues (1.73 ± 0.165) than that of in the corresponding noncancerous tissues (2.34 ± 0.145) (* *p* < 0.05.)

The result of qPCR test showed that the SESN2 mRNA expression was statistically lower in HCC tissues (1.73 ± 0.165) than that of in the corresponding noncancerous tissues (2.34 ± 0.145) (t = 2.759, *p* = 0.01, Fig. 1).

### Detection of protein expression of SESN2 by western blotting analysis

**Fig. 2:**
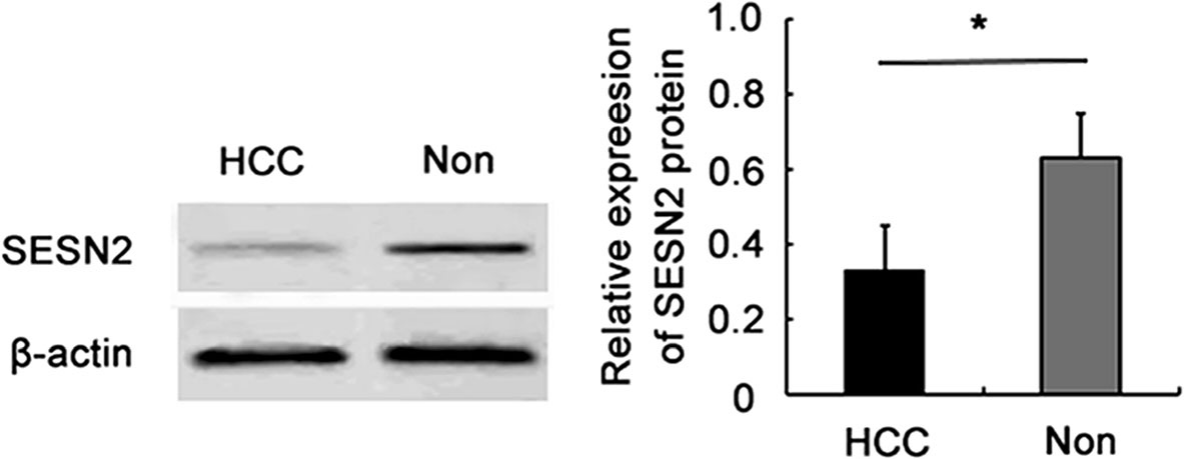
SESN2 expression detected by western blotting analysis in 15 HCC and noncancerous tissue samples. The expression level of SESN2 in HCC were significantly reduced comparing to that of in noncancerous tissues (* *p* < 0.05)

Western blotting analysis in 15 HCC and corresponding noncancerous tissue samples was performed to evaluate the protein expression of SESN2. The results showed the similar trend in qPCR test that SESN2 expression in HCC tissues was statistically lower than that of in noncancerous tissues (Fig. 2).

### Measurement of protein expression of SESN2 by IHC analysis

**Fig. 3:**
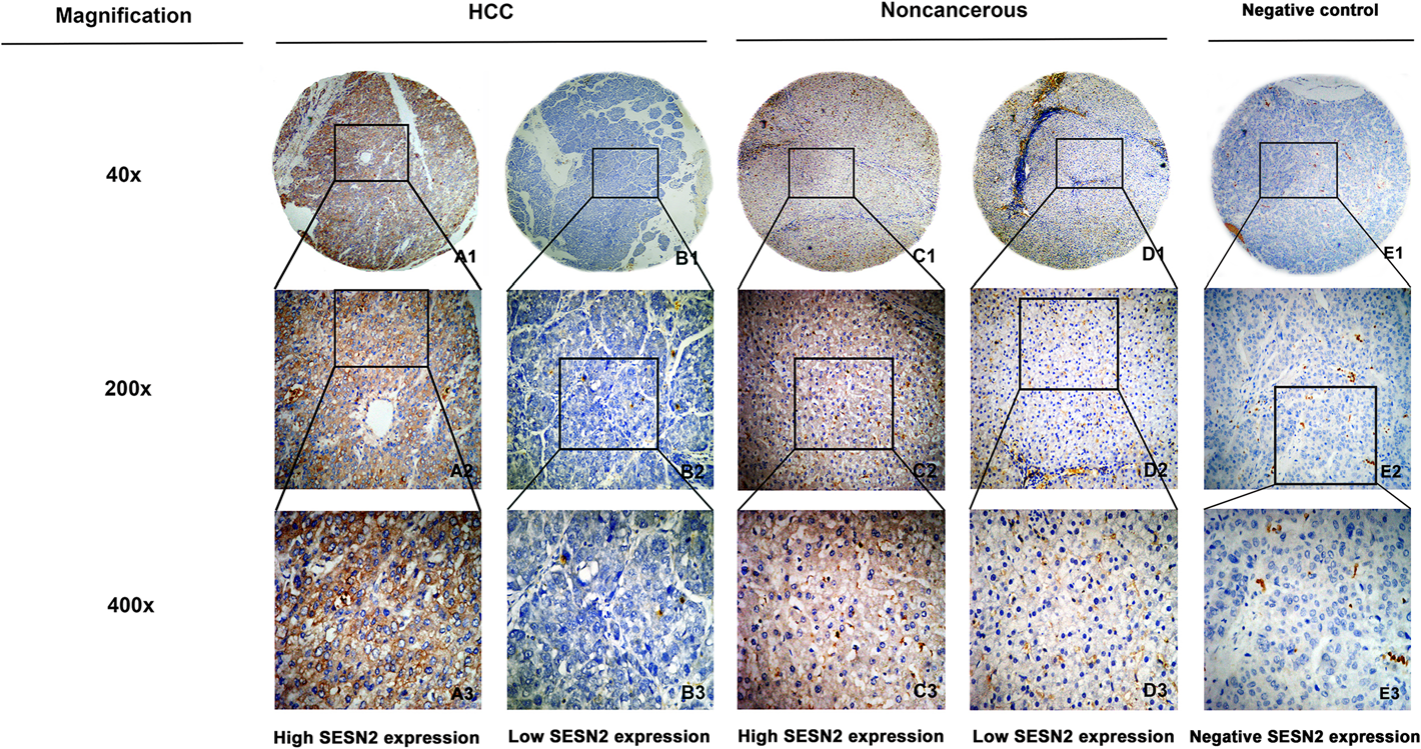
Immunohistochemistry (IHC) analysis in HCC tissue microarray (TMA) was executed to detect SESN2 expression. A1-A3 High staining of SESN2 in HCC sample. B1-B3 Low staining of SESN2 in HCC sample. C1-C3 High staining of SESN2 in noncancerous sample. D1-D3 Low staining of SESN2 in non-cancerous sample. E1-E3 Negative staining of SESN2 in HCC sample

The data of IHC analysis demonstrated that high SESN2 expression was observed in 38 of 100 HCC tissue samples (38.0%), whereas 71 of 100 noncancerous normal tissue cases (71.0%) showed high SESN2 expression. In HCC tissues,the SESN2 protein expression level was significantly reduced compared to noncancerous tissues (*p* < 0.05). Positive staining of SESN2 was mainly located in the cytoplasm of HCC cells (Fig. 3).

### Association between SESN2 protein expression and clinicopathological characteristics of HCC

The association between high SESN2 protein expression and the clinicopathological characteristics of 100 HCC patients are shown in Table 1. SESN2 expression was associated with HBV infection (*p* = 0.019), HCV infection (*p* = 0.001) and lymph node metastasis (*p* = 0.033). In contrast, no statistical association was noticed between SESN2 expression and other clinical items, including the gender, age, liver cirrhosis, tumor size, distant metastasis, and TNM stage (Table 1).

**Table 1:** Relationship of high SESN2 expression with clinicopathological characteristics in HCC

Groups	No.	SESN2		*χ* ^2^	*p* value
		+	%		
Gender					
Male	83	31	37.3	0.088	0.767
Female	17	7	41.2		
Age (years)					
< 60	58	18	31.0	2.844	0.092
≥ 60	42	20	47.6		
Hepatitis B virus infection					
Positive	41	10	24.4	5.463	0.019*
Negative	59	28	47.5		
Hepatitis C virus infection					
Positive	52	31	59.6	21.484	0.001*
Negative	48	7	14.6		
Liver cirrhosis					
Positive	58	21	36.2	0.189	0.664
Negative	42	17	40.5		
Tumor size (cm)					
> 5	57	19	33.3	1.225	0.268
≤ 5	43	19	44.2		
Lymph node metastasis					
Positive	31	7	22.6	4.534	0.033*
Negative	69	31	44.9		
Distant metastasis					
Positive	12	4	33.3	0.126	0.723
Negative	88	34	38.6		
TNM stage					
Stage I-II	41	17	41.5	0.354	0.552
Stage III-IV	59	21	35.6		

*p < 0.05

### Survival analysis

**Fig. 4:**
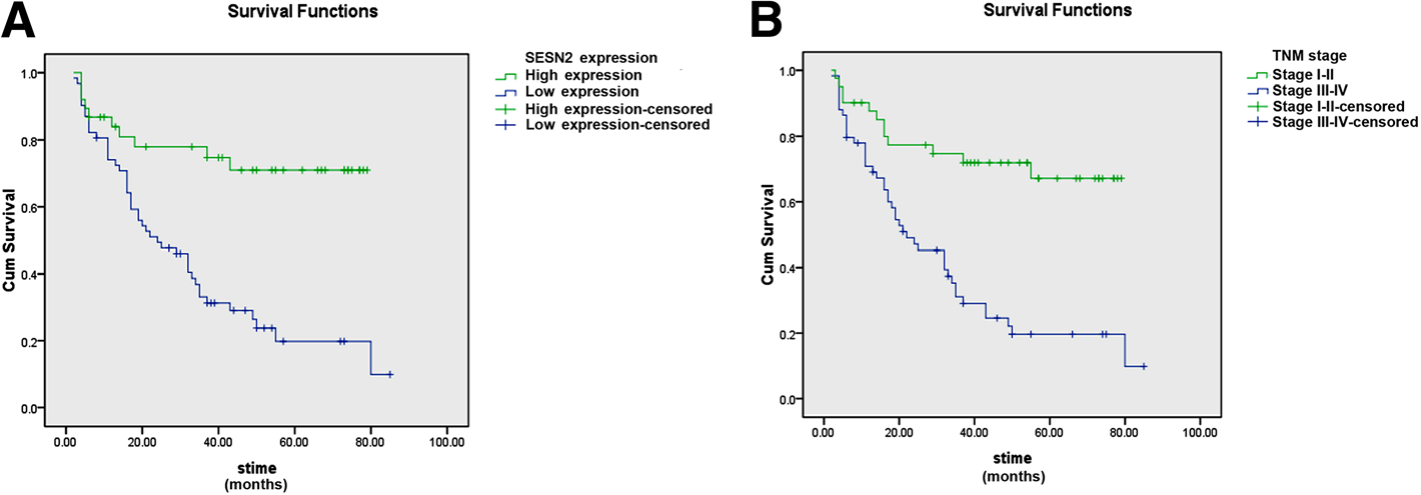
The Kaplan-Meier curve was drawn to illustrate two independent prognostic factors for overall survival of 100 HCC patients. **a** Overall survival rate in patients with low SESN2 expression was significantly lower than that in patients with high SESN2 expression. **b** Overall survival rate in patients with early TNM stage was significantly higher than that in patients with advanced TNM stage

For survival analysis, univariate test was firstly performed and the results stated that the prognosis of 100 HCC patients was significantly correlated with the SESN2 expression level (*p* = 0.001), HCV infection (*p* = 0.036), lymph node metastasis (*p* = 0.001), distant metastasis (*p* = 0.003), and TNM stage (*p* = 0.001). Subsequently, multivariate test further screened that SESN2 expression (*p* = 0.003) and TNM stage (*p* = 0.003) may serve as two independent prognostic factors for overall survival of 100 HCC patients in the present research (Table 2). Kaplan-Meier curves explained that HCC patients with high SESN2 protein expression and early TNM staging had significantly more favorable survival times (Fig. 4).

**Table 2:** Survival analysis by univariate and multivariate methods to identify prognostic factors in HCC

	Univariate analysis			Multivariate analysis		
	HR	*p* value	95% CI	HR	*p* value	95% CI
SESN2 expression						
High versus Low	0.271	0.001*	0.136–0.542	0.308	0.003*	0.140–0.678
Gender						
Male versus Female	1.140	0.732	0.539–2.414			
Age (years)						
< 60 versus ≥60	1.630	0.084	0.937–2.838			
Hepatitis B virus infection						
Positive versus Negative	1.111	0.900	0.526–1.539			
Hepatitis C virus infection						
Positive versus Negative	1.799	0.036*	1.039–3.114	0.940	0.851	0.493–1.793
Liver cirrhosis						
Positive versus Negative	1.137	0.644	0.660–1.959			
Tumour size (cm)						
> 5 versus ≤5	1.412	0.219	0.814–2.449			
Lymph node metastasis						
Positive versus Negative	2.795	0.001*	1.621–4.821	1.839	0.063	0.968–3.495
Distant metastasis						
Positive versus Negative	2.709	0.003*	1.417–5.179	0.925	0.846	0.421–2.033
TNM stage						
Stage I-II versus Stage III-IV	0.276	0.001*	0.144–0.527	0.352	0.003*	0.178–0.696

*p < 0.05

## Discussion

Sestrins are a family of highly conserved, stress-inducible genes that can protect cells against oxidative damage and oncogenic signaling [28]. Recently, one member of this family, SESN2, has received attention for acting as a tumor suppressor that can inhibit angiogenesis and promote apoptosis [12, 29]. This finding underscores the significance of illustrating the molecular mechanism by which SESN2 affects pathways for metabolism and survival. Sanli et al. reported that SESN2 could facilitate AMPK phosphorylation through a combination effect with a tumor suppressor, LKB1 [30] and increase enzyme auto-phosphorylation in breast cancer [21]. SESN2 expression inhibited cell growth and proliferation by suppressing mTOR through AMPK signaling modulation [15]. High expression of SESN2 was found to induce apoptosis through the AMPK/p38 signaling pathway in colon cancer cells [31]. The induction of SESN2 also enhanced the oxidative stress response and showed protective effectiveness in mice against tumor development through mTOR, and p53/p21-signaling network [32]. The above-mentioned information indicates that SESN2 has anti-oncogenic roles in several human cancers. The detailed relationship between SESN2 expression and clinicopathological significance of HCC deserve further exploration.

In the present study, the qPCR test indicated that the mRNA expression of SESN2 was critically reduced in HCC tissues compared with that in the corresponding noncancerous tissues. Moreover, the results of Western blotting and IHC analyses also demonstrated reduced protein expression of SESN2 in HCC cells relative to noncancerous cells. The above data are consistent with previous studies reporting the inhibited expression of SESN2 in several human cancers [19, 20]. Moreover, considerable associations of SESN2 expression and crucial clinicpathological characteristics in HCC exist, including HBV and HCV infection. We found that high SENS2 expression indicated negative lymph node metastasis. Wei et al. also described that low SESN2 expression is correlated with positive lymph node metastasis in colorectal cancer [33]. Our results agree with those obtained by Wei et al.; SESN2 expression could contribute to the restraint of certain malignant activities in HCC, such as tumor metastasis.

Thus far, studies concerning the association between SESN2 and cancer mortality in clinical samples are rare. In our present research, univariate and multivariate analyses illustrated that SESN2 expression and TNM stage were both correlated with the life span of HCC patients. In addition, the Kaplan-Meier curve analogously proved that HCC patients with low SESN2 expression encountered poor prognosis. The survival results were also in line with those of earlier studies, where high SESN2 expression prohibits tumor development and predicts favorable prognosis in cancers [15, 22, 33]. In all, SESN2 exerts significant anti-oncogenic effects, and high SESN2 expression substantially suspends malignant behavior that facilitates tumor development.

There are some limitations in this study that I need to address. For one thing, we failed to collect some important clinical items for HCC patients, including their AFP values, portal vein invasion statuses and tumor differentiation statuses, which are considered significant elements of the HCC etiology and development. We will perform more comprehensive data collection in our future studies. For another, the application of archived HCC samples may increase the bias in this retrospective observational study. Future studies that enroll more participants are needed to validate the present findings. Finally, the potential manner by which SESN2 influences the tumor microenvironment in HCC has not yet been explored. Future studies are of great importance to explore the mechanism by which SESN2 plays a role in HCC development.

In conclusion, this study first reported the differential expression of SESN2 in HCC. In particular, decreased SESN2 expression was observed in HCC. SESN2 expression was significantly associated with certain malignant behavior of HCC, including HBV/HCV infection and lymph node metastasis. Thus, high SESN2 expression implicated advantageous prognosis in HCC patients. Our current research is valuable in exploring the characteristics of SESN2 in HCC development.

## Conclusion

This work is the first report on both the mRNA and protein expression of SESN2 in HCC. Differential SESN2 expression was detected in HCC and noncancerous tissue samples, and HCC patients with reduced SESN2 expression levels were prone to suffer positive lymph node metastasis. Moreover, high expression of SESN2 implied advantageous prognosis in HCC patients.

## Abbreviations

AFP: Alpha-fetoprotein
HBV: Hepatitis B virus
HCC: Hepatocellular carcinoma
HCV: Hepatitis C virus
IHC: Immunohistochemistry
JNK: c-Jun N-terminal kinase
PBS: Phosphate-buffered saline
TMA: Tissue microarray
